# Supplementary material for: Exploring the role of routine brain imaging in detecting asymptomatic brain metastases in first diagnosed HER2-positive breast cancer
Source: PLoS One. 2025 Oct 16;20(10):e0334610. doi: 10.1371/journal.pone.0334610 (PMC12530607; doi:10.1371/journal.pone.0334610)
Supplement: Attachment — Submitted filename: comments to changed Her2 Brain mets 1.25.docx [file pone.0334610.s004.docx]

Dear Reviewers,

Thank you for your great comments on our manuscript. We worked hard to revise the manuscript, and changed the whole statistics to include patients only with a brain MRI.

Reviewer #1: General Comments:
In this manuscript, the authors evaluate the utility of routine PET-CT and MRI for detecting asymptomatic brain metastases in HER2-positive breast cancer patients. The study addresses a clinically significant issue and provides valuable insights into the limited role of routine imaging in this population. The manuscript highlights the low detection rate of brain metastases and discusses alternative imaging strategies, including advanced HER2-specific modalities. This is an important contribution to the field of oncology imaging, particularly in optimizing cost-effective and targeted screening protocols.

However, there are several areas that require major revisions to improve the clarity, methodological transparency, and overall impact of the manuscript. Key concerns include the lack of detailed imaging protocols (e.g., MRI acquisition techniques), the justification for using PET-CT in the assessment of brain metastases, and a more robust discussion of limitations and implications for clinical practice. Addressing these issues is essential to strengthen the manuscript's scientific rigor and clinical relevance.

Specific comments:
1. Comment on the Use of PET-CT
In the Methods and Results sections, it is mentioned that PET-CT was used for the evaluation of brain metastases. However, FDG PET-CT is known to be inferior to contrast-enhanced MRI in detecting metastatic brain tumors (1) and generally not suitable for detecting brain tumors or metastatic brain lesions. Please clarify the purpose of using PET-CT in this study and how its findings are interpreted. Additionally, elaborate on the impact of PET-CT results on the overall conclusions of the study. Thanks for your comments. We strongly thought about it and decided to follow your suggestion in point 3.

2. Comment on the Imaging Protocol for Contrast-Enhanced MRI
In the Results section, it is stated that brain metastases were detected using MRI; however, the specific imaging protocol for contrast-enhanced MRI is not provided. Studies have shown that 3D contrast-enhanced MRI is superior to 2D methods for detecting metastatic brain lesions (2). Furthermore, the ability to detect metastatic brain tumors in contrast-enhanced MRI scans depends on the imaging device (1.5T vs. 3T, etc.), the dose of contrast agent, and other factors (3) (4). Providing detailed information about the MRI protocol (e.g., 2D or 3D imaging, slice thickness, etc.) would enhance the reliability and interpretability of the results. Thanks for the comment. We tried to add all the missing stuff.

3. Suggestion to Limit the Analysis to MRI-Only Patients
For the diagnosis of metastatic brain lesions, it would be more consistent and reliable to limit the analysis to patients who underwent MRI, rather than including those evaluated with PET-CT. I recommend reconsidering the inclusion criteria and presenting results based solely on MRI findings. We followed your suggestion and are happy, that this recreation made the paper to a better one. Thanks again for your thoughtful comments.


1. Krüger S, Mottaghy FM, Buck AK, Maschke S, Kley H, Frechen D, et al. Brain metastasis in lung cancer. Comparison of cerebral MRI and 18F-FDG-PET/CT for diagnosis in the initial staging. Nuklearmedizin. 2011;50(3):101-6.
2. Kakeda S, Korogi Y, Hiai Y, Ohnari N, Moriya J, Kamada K, et al. Detection of brain metastasis at 3T: comparison among SE, IR-FSE and 3D-GRE sequences. European Radiology. 2007;17(9):2345-51.
3. Ochi T, Taoka T, Matsuda R, Sakamoto M, Akashi T, Tamamoto T, et al. Comparison between Two Separate Injections and a Single Injection of Double-dose Contrast Medium for Contrast-enhanced MR Imaging of Metastatic Brain Tumors. Magnetic Resonance in Medical Sciences. 2014;13(4):221-9.
4. Jensen LG, Nath SK, Murphy KT, Alksne JF, Mundt AJ, Lawson JD. Difference in Number of Brain Metastases Detected using 1.5T and 3T MRI in Patients Treated with Stereotactic Radiosurgery. International Journal of Radiation Oncology, Biology, Physics. 2011;81(2):S300.

Reviewer #2: In this article, Leser et al. evaluated the relevant topic of proactive brain imaging in patients with HER2-positive breast cancer. The authors analyzed a cohort of 153 patients with newly diagnosed HER2-positive breast cancer, identifying only 4 patients with brain metastases. However, several significant limitations render this study unsuitable for publication:

-Patient Cohort Selection: The authors included patients at any disease stage, rather than focusing on those with advanced-stage disease, which is the clinical setting where brain imaging is typically considered, undermining the relevance of the findings. Furthermore, the disease stage of the patients with brain metastases is not reported. We added the stages of the patients with brain metastases and will follow your suggestion in further prospective studies. Thank you for the suggestion.

-Screening Methodology: It appears that PET/CT scans were used for brain metastasis screening in some cases, which constitutes a major methodological flaw. As the authors themselves acknowledge, PET/CT is inadequate for detecting brain metastases. Indeed, all identified brain metastases were diagnosed via MRI, further highlighting the inappropriate reliance on PET/CT. Thanks for your comment. Because of your suggestion and the one from the other reviewer we limit the analysis to MRI-only patients.

-Small Sample Size: The extremely low number of cases with brain metastases (n=4) makes the associations reported in the study highly unstable. Additionally, disease stage—an expectedly strong predictor of brain involvement—could heavily confound any observed associations. Thank you for your thoughtful comment. Because of your comment, we looked on the correlation between stage and lymphnode status and it was significant. So, thanks again to improve the results with your impact.

I hope that the changes meet your expectations, and are bringing our manuscript closer to publishing in your manuscript.

Greets
