# Supplementary material for: Exploring the role of routine brain imaging in detecting asymptomatic brain metastases in first diagnosed HER2-positive breast cancer
Source: PLoS One. 2025 Oct 16;20(10):e0334610. doi: 10.1371/journal.pone.0334610 (PMC12530607; doi:10.1371/journal.pone.0334610)
Supplement: Attachment — Submitted filename: Comments.docx [file pone.0334610.s003.docx]

In this article, the author claimed the clinical value of routine brain imaging for all asymptomatic HER2-positive patients may be limited. The patients with a higher likelihood of brain metastasis development, such as those with additional metastatic sites or certain molecular markers, might benefit more from regular imaging. In addition, HER2-specific PET imaging may improve early detection and reduce unnecessary imaging in lower-risk patients, supporting a more efficient and clinically valuable strategy in managing HER2-positive breast cancer patients at risk for brain metastasis. This is a very interesting study. But there are still a few points that confuse me.

1. In the result, the author claimed that “ The majority of tumors were classified as size category 1 (n = 469, 445.54%), while 3248 (2931.16%) were classified category 2, 13 9(8.26%) were classified category 3, and 22 19 (174.35%) were classified as category 4. “ Please supplement the grading method in the methodology section.
2. Among the 110 patients included, how many patients actually had brain metastases, and was there any situation of missed diagnosis by MRI?
3. In Discussion section, the potential value of HER2-PET imaging application has been discussed in the text before and after the Discussion. Please merge them.
4. In Discussion section, The author extensively discusses “Ki67 Index and Tumor Characteristics”, “Hormone Receptor Status and Brain Metastasis”, “Implications for Routine Imaging in Asymptomatic HER2-Positive Patients”. It is suggested that the significance of referring to related molecular indicators to guide patients to choose brain MRI should be emphasized.
