# Supplementary material for: Exploring the role of routine brain imaging in detecting asymptomatic brain metastases in first diagnosed HER2-positive breast cancer
Source: PLoS One. 2025 Oct 16;20(10):e0334610. doi: 10.1371/journal.pone.0334610 (PMC12530607; doi:10.1371/journal.pone.0334610)
Supplement: Attachment — Submitted filename: Comments Reviewer4.25.docx [file pone.0334610.s006.docx]

Reviewer #3: dear authors, I have no more comments, thanks for working on the revisions.dear authors, I have no more comments, thanks for working on the revisions.dear authors, I have no more comments, thanks for working on the revisions.dear authors, I have no more comments, thanks for working on the revisions.dear authors, I have no more comments, thanks for working on the revisions.dear authors, I have no more comments, thanks for working on the revisions.

Thanks for your comment

Reviewer #4: There are some significant flaws to be addressed with the manuscript.
The patient population must be better defined, why were males excluded,(we as gynecologists, are only working with breast cancer in women) did all these women undergo resection with curative intent, how many received neo-adjuvant systemic therapy (consider standard of care for women with Her2 positive disease).(All women without metastases were treated with a neoadjuvant chemotherapy and after with a curative resection)
Was there any evidence of extra-cranial metastatic disease. Included (13 patients with metastases other than brain)
At what time point was the MRI done? at diagnosis? (yes, it was done at diagnosis, newly metioned) Were these women symptomatic? All were asymptomatic Describe the size and number of lesions that were detected in these 4 women. added
Please define the tumor characteristics using TNM classification, for size and nodal status, ot explain the system used. TNM was used
DCIS was an exclusion criteria (obviously as by definition these cannot have metastatic disease), yet we are told in great detail how many women had DCIS, not relevant. deleted
The spelling or Oestrogen/estrogen hormon/hormone status needs to be consistent throughout the paper. changed

Unfortunately the very small sample size (4 brain tumours detected) makes this study of limited utility.

Thanks for your comments and making the paper a better one.

Reviewer #5: In this article, the author claimed the clinical value of routine brain imaging for all asymptomatic HER2-positive patients may be limited. The patients with a higher likelihood of brain metastasis development, such as those with additional metastatic sites or certain molecular markers, might benefit more from regular imaging. In addition, HER2-specific PET imaging may improve early detection and reduce unnecessary imaging in lower-risk patients, supporting a more efficient and clinically valuable strategy in managing HER2-positive breast cancer patients at risk for brain metastasis. This is a very interesting study. But there are still a few points that confuse me.
1.In the result, the author claimed that “ The majority of tumors were classified as size category 1 (n = 469, 445.54%), while 3248 (2931.16%) were classified category 2, 13 9(8.26%) were classified category 3, and 22 19 (174.35%) were classified as category 4. “ Please supplement the grading method in the methodology section. updated
2.Among the 110 patients included, how many patients actually had brain metastases, and was there any situation of missed diagnosis by MRI? 4 patients had brain metastases, best of our knowledge no one was missed
3.In Discussion section, the potential value of HER2-PET imaging application has been discussed in the text before and after the Discussion. Please merge them. changed
4.In Discussion section, The author extensively discusses “Ki67 Index and Tumor Characteristics”, “Hormone Receptor Status and Brain Metastasis”, “Implications for Routine Imaging in Asymptomatic HER2-Positive Patients”. It is suggested that the significance of referring to related molecular indicators to guide patients to choose brain MRI should be emphasized. Mentioned in future perspectives

Thanks for your comments and making the paper a better one.
